# Clinical and histological corelations in chronic viral hepatitis C

**DOI:** 10.1186/1471-2334-13-S1-P52

**Published:** 2013-12-16

**Authors:** Mirela Indries

**Affiliations:** 1 Municipal Clinical Hospital "Dr. Gabriel Curteanu" Oradea, Romania

## Background

Infection with hepatitis C virus (HCV) is a major public health problem worldwide due to asymptomatic evolution to cirrhosis and liver cancer. In patients with chronic liver disease, the precise definition of the stage of liver fibrosis is important for prognosis and monitoring of the evolution of liver disease and the outcome of antiviral therapy in chronic HCV infection. Activation of hepatic stellate cells (HSCs) and transformation into myofibroblasts is the central event in liver fibrosis.

## Methods

The aim of this study was to perform clinical and histological correlations in patients with chronic HCV infection. The study was based on clinical and laboratory data, including liver histology, recorded in a group of patients admitted to the Emergency County Hospital Oradea, Department of Clinical Gastroenterology.

## Results

The study included 137 patients aged 19 to 69 years. The balance in terms of gender representation was slightly inclined towards the female gender with a percentage of 58.76%.

Approximately two thirds of patients (66.06%) had ages between 36 and 55, slightly higher for the age group 46-55 years (33.58% versus 32.48%). We examined between 4 and 24 vein spaces, with an average of 7.8 portal spaces and a standard derivation of 3.82, mostly from 4 to 11 areas examined. Most patients were at their first liver biopsy (88.32%), 9.49% at the second biopsy, and 5 patients (1.82%) had previously undergone 3 to 4 punctures. The following fibrosis stages were recorded: F0 6.2%, F1: 40.51%, F2 36.5 %, and F3 16.79%.

Most (24.45%) had portal fibrosis without septa and mild inflammatory activity, the next had portal fibrosis with rare septa of fibrosis and mild activity (10.22%) and 6.2% with severe activity.

The age group over 66 years did not present cases of chronic hepatitis without fibrosis and the age group up to 25 years did not present cases of chronic hepatitis with fibrosis F3. For the age group 46-55 years predominate F3 (6.2%) and F1 (14.23%), F2 36-45 (12.77%).

## Conclusion

For the clinician, the fibrosis progression is the most faithful parameter of the evolution of liver disease.

